# Pressure for drug development in lysosomal storage disorders – a quantitative analysis thirty years beyond the US orphan drug act

**DOI:** 10.1186/s13023-015-0262-5

**Published:** 2015-04-18

**Authors:** Konstantin Mechler, William K Mountford, Georg F Hoffmann, Markus Ries

**Affiliations:** Department of Child and Adolescent Psychiatry and Psychotherapy, Pediatric Psychopharmacology, Central Institute of Mental Health, Medical Faculty Mannheim, University of Heidelberg, Mannheim, Germany; Clinical Research Program, University of North Carolina Wilmington, Wilmington, NC USA; Pediatric Neurology and Metabolic Medicine, Center for Rare Diseases, Center for Pediatric and Adolescent Medicine, Heidelberg University Hospital, Im Neuenheimer Feld 430, Heidelberg, D-69120 Germany

**Keywords:** Orphan disease, Drug development, Small clinical trials

## Abstract

**Background:**

Lysosomal storage disorders are a heterogeneous group of approximately 50 monogenically inherited orphan conditions. A defect leads to the storage of complex molecules in the lysosome, and patients develop a complex multisystemic phenotype of high morbidity often associated with premature death. More than 30 years ago the Orphan Drug Act of 1983 passed the United States legislation intended to facilitate the development of drugs for rare disorders.

We directed our efforts in assessing which lysosomal diseases had drug development pressure and what distinguished those with successful development and approvals from diseases not treated or without orphan drug designation.

**Methods:**

Analysis of the FDA database for orphan drug designations through descriptive and comparative statistics.

**Results:**

Between 1983 and 2013, fourteen drugs for seven conditions received FDA approval. Overall, orphan drug status was designated 70 times for 20 conditions. Approved therapies were enzyme replacement therapies (N = 10), substrate reduction therapies (N = 1), small molecules facilitating lysosomal substrate transportation (N = 3). FDA approval was significantly associated with a disease prevalence higher than 0.5/100,000 (p = 0.00742) and clinical development programs that did not require a primary neurological endpoint (p = 0.00059). Orphan drug status was designated for enzymes, modified enzymes, fusion proteins, chemical chaperones, small molecules leading to substrate reduction, or facilitating subcellular substrate transport, stem cells as well as gene therapies.

**Conclusions:**

Drug development focused on more common diseases. Primarily neurological diseases were neglected. Small clinical trials with either somatic or biomarker endpoints were successful. Enzyme replacement therapy was the most successful technology. Four factors played a key role in successful orphan drug development or orphan drug designations: 1) prevalence of disease 2) endpoints 3) regulatory precedent, and 4) technology platform. Successful development seeded further innovation.

## Background

### Lysosomal storage disorders

Lysosomal storage disorders (LSDs) are a clinically heterogeneous group of more than 40 inherited orphan conditions. Their prevalence was determined in various surveys to 13 per 100,000 live births (=1 in 7700) in Australia [1], 14 per 100,000 live births (=1 in 7143) in the Netherlands [2], 7.6 per 100,000 live births (=1 in 13,158) in British Columbia [3], and 25 per 100,000 live births (=1 in 4000) in Portugal [4]. These diseases share a common pathobiochemical leitmotiv: a genetic defect leads to the storage of complex non-metabolized molecules in the lysosome. The biochemical identification of this storage material led to the traditional classification of LSDs into lipidoses (including sphingolipidoses), mucopolysaccharidoses (MPSs), glycogenosis, cystinosis, mucolipidoses, oligosaccharidoses, and neuronal ceroid lipofuscinoses. Despite the common mechanism, each of these disorders is distinct with its own pathophysiology and clinical presentation. LSDs are in general multisystemic, progressive disorders of significant morbidity with decreased life-expectancy that can manifest within a heterogeneous somatic and neurological spectrum such as hydrops fetalis, dysmorphism, dysostosis multiplex, hepatosplenomegaly, central nervous system disease, ophthalmologic, cardiovascular, renal, or cutaneous disease features [5].

### U.S. orphan drug act

Whereas the impetus to develop drugs is driven by unmet medical need, from a pharmaceutical company's perspective, this is predicated on returns on investment, ultimately influenced by the likelihood of success in clinical trials and commercialization. The US Orphan Drug Act passed in 1983 with the goal to stimulate the investment into the development of medicines for rare diseases through various incentives, such as seven years' marketing exclusivity, tax credit for 50% of clinical trial costs, protocol assistance, Food and Drug Administration fee waiver, and orphan products grants program [6]. By December 2013, a total of 456 orphan indications were approved by the FDA [7].

The key factors for successful drug development of therapies for lysosomal storage disorders have not been systematically analyzed. We therefore directed our efforts in assessing which lysosomal diseases had drug development pressure and what distinguished those with successful development and approvals from diseases not treated or with no orphan drug designations. Neurological endpoints were a focus of this study because many lysosomal storage disorders are neurological conditions. We analyzed whether disease prevalence, technology platforms, endpoints in clinical trials, and regulatory precedent were associated with successful drug development.

## Methods

### Data acquisition

We searched the FDA database for orphan drug designations with pertinent keywords for all lysosomal storage disorders at http://www.accessdata.fda.gov/scripts/opdlisting/oopd/. Start date of data entry was 01/01/1983. All data entries until 11/30/2013 were considered.

Epidemiological data on rare disorders were extracted from the Orphanet Report series [8]. Information on clinical studies were obtained from clinicaltrials.gov.

In order to account for publication bias, data on registration studies were obtained from the current FDA approved drug label accessed at http://www.accessdata.fda.gov/scripts/cder/drugsatfda/.

### Definitions

Pharmacological compounds were categorized into the following technology platforms based on their biochemical and therapeutic characteristics: enzyme replacement therapy, substrate reduction therapy, small molecules facilitating intracellular substrate transport, chemical chaperones, gene therapy, stem cell therapy, and others (such as adjunctive therapies). Regulatory precedent was defined as a drug approval by the FDA in the same or a clinically very similar disease, such as the different forms of mucopolysaccharidoses (MPSs). Time to FDA approval was defined as the time period from orphan drug designation until approval by the FDA.

### Statistical analysis

Characteristics from each of the identified pharmacological compounds were summarized using descriptive statistics. Continuous variables were summarized with mean, standard deviation, median, minimum and maximum values. Categorical variables were summarized with frequencies and percentages. Key compound characteristics, including requirement for neurological endpoints in clinical trials, regulatory precedent, disease prevalences (categorized either as <5/1,000,000 or ≥ 5/100,000) were compared for those compounds receiving orphan drug designation as well as receiving FDA approval to determine if associations existed. Tests for associations between categorical variables were performed using Fisher’s Exact Test as a result of low cell counts, where a two-sided p-value < 0.05 was considered statistically significant. All statistical analyses were performed using SAS Enterprise Guide version 9.1 (SAS, Cary, NC, USA).

## Results

### Successfully developed therapies

**Figure 1:**
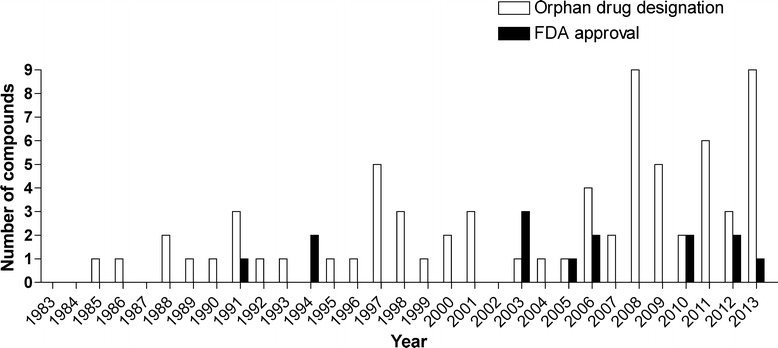
Number of orphan drug designations (open bars) and FDA approvals (full bars) for compounds intended to treat lysosomal storage diseases by year.

**Figure 2:**
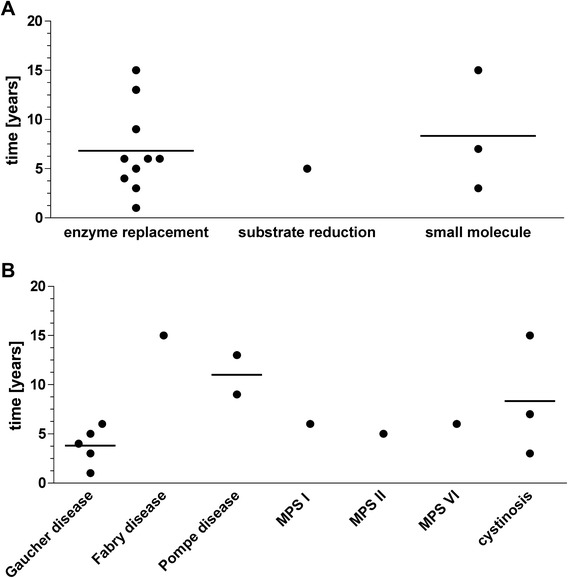
Time to approval of compounds intended to treat lysosomal storage diseases by **A)** technology platform and **B)** disease. Lines indicate means.

From 1983 until 2013, fourteen drugs for seven lysosomal storage disorders received FDA approval (Figure 1, Table 1). Two conditions had multiple drug approvals: Gaucher disease (N = 5) and cystinosis (N = 3). Five conditions had one FDA drug approval, respectively: Fabry disease, Pompe disease, MPS I, MPS II, and MPS VI (Figure 2). The first drug approved with orphan designation for a lysosomal storage disorder was alglucerase for Gaucher disease in 1991.

**Table 1:** **FDA approved compounds for lysosomal storage disorders, endpoints and design of clinical trials**

**Disease**	**Compound**	**Therapeutic class**	**Endpoints in pivotal studies**	**Primary neurological endpoints**	**Biomarker endpoints**	**Regulatory precedent**	**Pivotal trial design**	**Ref.**
MPS I	Laronidase	Enzyme	Forced vital capacity (% of predicted), 6 min walk distance	No	No	No	RCT, 26 weeks, N = 45, mean age 15.5 years (range 6 – 43 years)	[24]
MPS II	Idursulfase	Enzyme	Forced vital capacity (% of predicted), 6 min walk distance	No	No	Yes	RCT, 53 weeks, N = 96, mean age 14.2 years (range 5–31 years)	[25]
MPS VI	Galsulfase	Enzyme	12 min walk distance, 3 min stair climb test (stairs/min)	No	No	Yes	RCT, 24 weeks, N = 39, (age range 5–29 years)	[26]
Gaucher disease type I	Alglucerase	Enzyme	Liver and spleen volume change, Hematologic deficiencies, improved mineralization of bone, cachexia and wasting	No	Yes	No	OLT, 36 – 52 weeks, N = 13, mean age 20.3 years (range 7–42 years)	[27]
Gaucher disease	Imiglucerase	Enzyme	Anemia and thrombocytopenia, liver and spleen volume change, decreased cachexia	No	Yes	Yes	RCT, 26 weeks, N = 30, mean age 32.7 years (range 12 – 69 years)	[28]
Gaucher disease	Taliglucerase alfa	Enzyme	Hemoglobin concentration, platelet count, liver and spleen volume change	No	Yes	Yes	RCT, 36 weeks, N = 32, mean age 36.2 years (range 19 – 74 years)	[29]
Gaucher disease	Velaglucerase alfa	Enzyme	Hemoglobin concentration, platelet count, liver and spleen volume change	No	Yes	Yes	RCT, N = 25, 52 weeks, median age 25 years, (range 4–62 years)	[30]
Gaucher disease	Miglustat	Substrate reduction	Liver and spleen volume change, hemoglobin concentration, platelet count	No	Yes	Yes	OLT, 52 weeks, N = 28, mean age 44 years (range 22–69 years)	[31]
Fabry disease	Agalsidase beta	Enzyme	Reduction of GL-3 inclusions in capillary endothelium of kidney, heart and skin	No	Yes	No	RCT, 20 weeks, N = 58, mean age 30.2 years (range 16–61 years)	[32]
Pompe disease	Alglucosidase alfa	Enzyme (bioreactor size: 160 L)	Number of patients who died or needed invasive ventilator support	No	No	No	OLT, 52–106 weeks, N = 18, age range 1 month to 3.5 years	[33]
Pompe disease	Alglucosidase alfa	Enzyme (bioreactor size: 4000 L)	Forced vital capacity (% of predicted), 6 min walking distance	No	No	No	RCT, 78 weeks, N = 90, mean age 44.4 years (range 10–70 years)	[34]
Cystinosis	Cysteamine bitartrate IR	Small molecule	Serum creatinine, calculated creatinine clearance, growth (height)	No	Yes	No	OLT, N = 94, mean age 3.8 years	[35]
Cystinosis	Cysteamine ophtalmic solution	Small molecule	Corneal Cystine Crystal Score	No	Yes	No	OLT, N = 283 (three studies)	[36]
Cystinosis	Cysteamine bitartrate DR	Small molecule	White blood cell cystine	No	Yes	Yes	RCT, N = 43, mean age 12 years (range 6 – 26 years)	[37]

RCT – randomized controlled trial, OLT – open label trial, IR – immediate release, DR – delayed-release, GL-3 – globotriaosylceramide.

**Figure 3:**
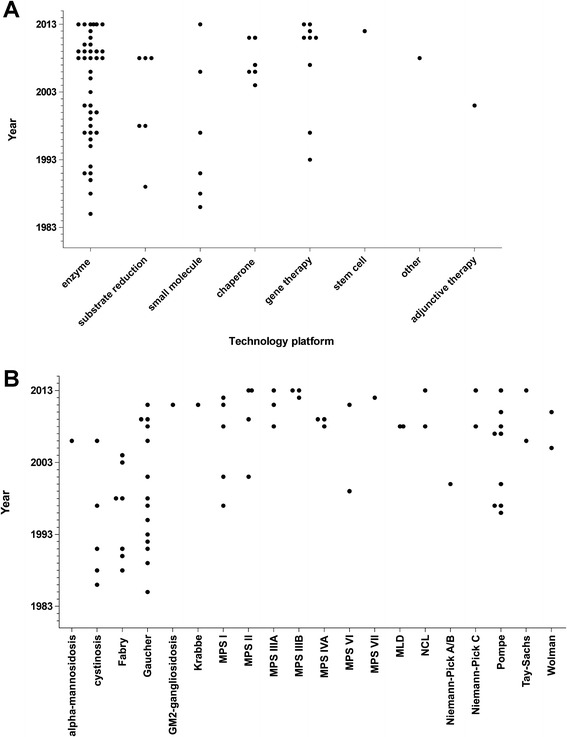
Year of orphan drug designation for compounds intended to treat lysosomal storage diseases. **A)** by technology platform. **B)** by disease.

**Figure 4:**
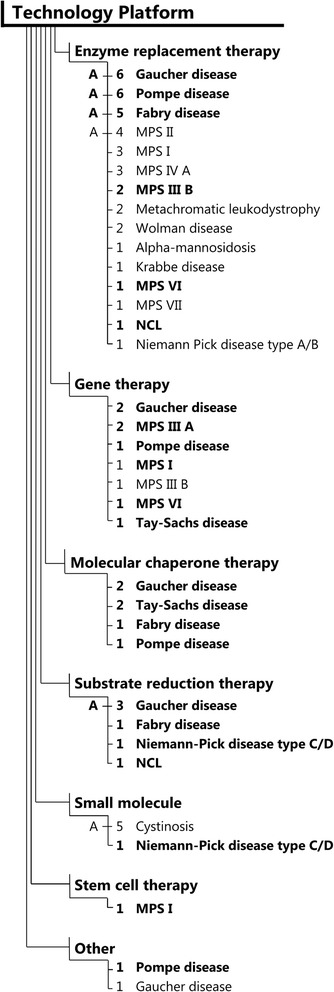
Orphan drug designations for compounds intended to treat lysosomal storage disorders by technology platforms and diseases. N indicates number of orphan drug designations granted. Bold indicates orphan drug designation across two or more technology platforms. “A” indicates disease with one or more FDA approved therapies in the particular technology platform.

Alglucerase had the first orphan drug designation which occurred in 1985. Most designations in a single year were granted in 2008 and 2013 with nine designations per year, respectively (Figures 1 and 3). From 1983 until 2013, orphan drug status was designated 70 times for 20 conditions of lysosomal storage disorders. Five diseases had a single orphan drug designation and 15 diseases had multiple orphan drug designations (Figure 4). Four designations were withdrawn. The mean (standard deviation) time between orphan drug designation and approval was 6.2 (3.9) years with a median of 5.5 years and a range of 1 – 15 years (N = 14). Figure 2 demonstrates the variability in time from orphan drug designation to approval for the various technology platforms and conditions.

### Factors associated with successful drug development for lysosomal storage diseases

#### 1) Prevalence of disease

The proportions of drugs receiving FDA approval and orphan drug designations were higher in rare diseases than in very rare diseases: of the 14 FDA approved drugs, 12 were for more frequent lysosomal storage diseases (prevalence ≥ 5/1,000,000) and only two were for less frequent conditions (prevalence < 5/1,000,000; p = 0.00742) (Table 2). Likewise, orphan drug designations were granted for 45 drugs developed to treat lysosomal storage disorders with a prevalence of ≥ 5/1,000,000, whereas only 25 drugs for less frequent diseases with a prevalence of < 5/1,000,000 received orphan drug designations, and in 17 very rare diseases, there was no drug in development at all (p < 0.0001).

**Table 2:** **Statistical analysis of clinical, regulatory, and epidemiological factors associated with a) FDA approval for compounds intended to treat lysosomal storage disorders and b) orphan drug designation**

**a)**			
**Characteristic**	**FDA approval (N = 14)**	**No FDA approval (N = 73)**	P-value*
Neurological endpoint	0 (0%)	34 (46.6%)	0.00059
No neurological endpoint	14 (100%)	39 (53.4%)	
Regulatory precedent	9 (64.3%)	31 (42.5%)	0.15411
No regulatory precedent	5 (35.7%)	42 (57.5%)	
Prevalence < 5/1,000,000	2 (14.3%)	40 (54.8%)	0.00742
Prevalence ≥ 5/1,000,000	12 (85.7%)	33 (45.2%)	
Orphan status designation	14 (100%)	56 (76.7%)	0.06229
No orphan status designation	0 (0%)	17 (23.3%)	
**b)**			
**Characteristic**	**Orphan status (N = 70)**	**No orphan status (N = 17)**	P-value*
Neurological endpoint	19 (27.1%)	15 (88.2%)	< 0.0001
No neurological endpoint	51 (72.9%)	2 (11.8%)	
Regulatory precedent	39 (55.7%)	1 (5.9%)	0.00022
No regulatory precedent	31 (44.3%)	16 (94.1%)	
Prevalence < 5/1,000,000	25 (35.7%)	17 (100%)	< 0.0001
Prevalence ≥ 5/1,000,000	45 (64.3%)	0 (0%)	

*P-values are from Fisher’s Exact Test.

#### 2) Neurological vs somatic endpoints and biomarkers

Neurological endpoints were statistically significant factors for successful drug development and orphan drug designations (Table 2). Of the approved 14 compounds, no clinical development program had a primary neurological endpoint. Most, i.e. 51/70 (72.9%), orphan drug designations were sought for diseases that would, by nature of the disease, not require a neurological endpoint in clinical studies. In contrast, most conditions without an orphan drug designation, i.e. 15/17 (88.2%), would require a clinical trial with a neurological endpoint. Three successful orphan drug programs were based on biomarkers, i.e. in Gaucher disease, Fabry disease, and cystinosis (Table 1). The time to FDA approval for these three conditions tended to be shorter than for conditions with clinical endpoints, although the difference was not statistically different.

#### 3) Regulatory precedent

Regulatory precedent, i.e. a drug approval in the same or a clinically very similar disease (e.g. the MPS group), was a statistically significant factor for orphan drug designation in lysosomal storage diseases (Table 2). Most orphan drug designations, i.e. 39/70 (55.7%) had a regulatory precedent whereas, accordingly, there was no regulatory precedent for the majority of lysosomal storage diseases without orphan drug status, i.e. 16/17 (94.1%, p = 0.00022).

#### 4) Technology platforms

Approved therapies were enzyme replacement therapies (N = 10), small molecules (N = 3), and substrate reduction therapies (N = 1) as shown in Table 1. The most frequent orphan drug designations were enzymes of various sources and modified enzymes (N = 40), gene therapies (N = 9), small molecules (N = 6), substrate reduction therapies (N = 6), chaperones (N = 6), stem cell therapy (N = 1), and others (N = 2) as shown in Figure 4.

## Discussion

In the last three decades from 1983 until 2013, fourteen drugs for seven lysosomal storage disorders received FDA approval.

There were four factors that played a key role in successful orphan drug development or orphan drug designations: 1) prevalence of disease 2) endpoints 3) regulatory precedent, and 4) technology platform. These data demonstrate that the efforts in drug development were directed towards more common diseases. Primarily neurological diseases were neglected, and clinical trials utilized either somatic or biomarker endpoints. Clinical studies were mainly small clinical trials. Enzyme replacement therapy was the most successful technology in the last three decades followed by small molecules and substrate reduction.

One may think that the successful development of a therapy would stop further activities in the area. The contrary seems to be the case: innovation seeds innovations and success leads to more development pressure. Once a drug is approved, further orphan drug designations follow on as illustrated in Figure 2B. Enzyme replacement therapy for non-neurological Gaucher disease was the condition that seeded and orthodromically drove innovation. In addition to being the first, Gaucher disease also has the most approved therapies and most orphan drug designations. This may not be a coincidence, because Gaucher disease is a more frequent condition and the FDA approval was based on biomarkers as well as visceral endpoints which show timely and substantial treatment effects. Biomarker based programs, such as Gaucher disease and cystinosis tended to have shorter timelines from orphan drug designation to FDA approval.

Neurological endpoints appear to be problematic. Often, the natural history of the neurological disease is ill defined, validated quantitative endpoints across languages are not available or the treatment does not address the neurological manifestation of the disease as exemplified by two studies of enzyme replacement therapy and substrate reduction therapy in neuronopathic Gaucher disease [9,10]. The fact that neurological diseases received less attention is obviously not intentional. It is due to the types of drugs in development, and in particular, their mechanisms of action, and their ability to target sites of pathology across the blood brain barrier.

Regulatory precedent, i.e. the approval of a compound in the same disease or similar disease group, sets the pathway for successful downstream drug development in the same or a very similar condition. In the case of mucopolysaccharidoses (MPS I, II, and VI), clinical trials shared almost identical endpoints across programs, which may have facilitated the design of pivotal studies as the true validation of clinical endpoints in single rare diseases is complicated by the small size of available study populations and the slowly progressive nature of most diseases [11-13]. Likewise, the dialogue with the FDA becomes easier once the agency has become familiar with similar questions from a previous, successful drug development program. Interestingly, although approvals were focused on endpoints documenting the initial therapeutic response, the trials which have led to regulatory approval have not addressed the lifetime requirement for treatment and maintenance regimen.

Most approved and designated orphan drugs were enzymes. Substituted enzymes tend to work well on somatic endpoints and biomarkers as demonstrated in the successful clinical development programs outlined in Table 1, but the effect of enzyme replacement therapy is mainly compromised by late initiation of treatment, immune reactions against the therapeutic protein as well as incomplete accessibility of certain tissues by the protein such as skeletal muscle, bone, and especially brain [14-17]. Dose and frequency of enzyme administration are further important questions [18]. These shortcomings will direct future research.

Other successful approvals were small molecules and substrate reduction. If a lysosomal storage disease is of low prevalence and primarily a neurological condition our data would suggest low development pressure and low probability of success. This is illustrated by programs such as Niemann-Pick disease type C and Batten disease programs which neither led to the detection of strong therapeutic effects size or to FDA approval [19,20].

What must be done to address unmet needs for ultra-rare and neuronopathic lysosmal storage disorders? Various traditional and innovative technology platforms are being tested in human. As such, clinicaltrials.gov currently lists more than 70 open interventional clinical trials for lysosomal storage disorders studying enzymes, modified enzymes, substrate reduction, intrathecal drug delivery, chaperone therapy, hematopoetic stem cell transplantation, small molecules and gene therapy [21]. The results of this work will provide further insight into the ability to cure CNS diseases. Drug development in neuronopathic lysosomal storage disorders may be facilitated through the availability of better instruments assessing neurological and behavioral functions in a standardized way as proposed in the NIH toolbox [22]. Quantitative natural history studies are indispensable for a better understanding of the disease, design of clinical trials and the assessment of potential treatment effects. Biomarker development and better access to biomarker-based approvals as in Gaucher disease and cystinosis may be other points of consideration [23]. All the 14 FDA-approved compounds were developed by eight mainly small, specialized biopharmaceutical companies which have, in general, experienced sustainable economic growth over time.

### Limitations of this analysis

First, the designation of a compound as an orphan drug was considered a surrogate for the intent to develop a drug for a disease. Due to patent considerations, not all manufacturers may seek orphan drug designation by the FDA and information may therefore not be transparent. Second, time to approval may not reflect true development process because the time of orphan designation may be arbitrary in the drug development process. Third, the European Medicine Agency data were not formally analyzed, mainly because orphan legislation was introduced much later (2000) and the database is therefore less comprehensive. As drug development for orphan conditions is a global effort, and as the EMA orphan drug designations show similar trends (data not shown) the formal analysis of the FDA data and their impact for patients around the world are considered generalizable.

## Conclusions

Since the introduction of the US orphan drug act in 1983 until 2013, 14 orphan drugs, mostly enzyme replacement therapies, were developed for lysosomal storage disorders. Drug development was driven by more frequent conditions and diseases with somatic or biomarker endpoints sharing a similar pathway to registration. Successful development seeded further innovation.

## Abbreviations

FDA: Food and Drug administration
EMA: European Medicines Agency
FD: Fabry disease
GD: Gaucher disease
MPS: Mucopolysaccharidosis
NIH: National Institutes of Health
